# *Aedes aegypti* Mosquito Probing Enhances Dengue Virus Infection of Resident Myeloid Cells in Human Skin

**DOI:** 10.3390/v16081253

**Published:** 2024-08-05

**Authors:** Priscila M. S. Castanha, Sasha R. Azar, Jason Yeung, Megan Wallace, Gwenddolen Kettenburg, Simon C. Watkins, Ernesto T. A. Marques, Nikos Vasilakis, Simon M. Barratt-Boyes

**Affiliations:** 1 Department of Infectious Diseases and Microbiology, School of Public Health, University of Pittsburgh, Pittsburgh, PA 15261, USA; pmd35@pitt.edu (P.M.S.C.); mew201@pitt.edu (M.W.); gkettenburg@uchicago.edu (G.K.); marques@pitt.edu (E.T.A.M.); 2 Center for Tissue Engineering, Department of Surgery, Houston Methodist Research Institute, Houston Methodist Hospital, Houston, TX 77030, USA; srazar@houstonmethodist.org; 3 Department of Pathology, University of Texas Medical Branch, Galveston, TX 77555-0609, USA; 4 Department of Biochemistry, Cellular and Molecular Biology, University of Texas Medical Branch, Galveston, TX 77555-0645, USA; jayeung@utmb.edu; 5 Center for Biologic Imaging, University of Pittsburgh, Pittsburgh, PA 15261, USA; simon.watkins@pitt.edu; 6 Department of Immunology, School of Medicine, University of Pittsburgh, Pittsburgh, PA 15213, USA; 7 Aggeu Magalhaes Institute, Oswaldo Cruz Foundation, Recife 50.740-465, Pernambuco, Brazil; 8 Center for Vector-Borne and Zoonotic Diseases, University of Texas Medical Branch, Galveston, TX 77555-0609, USA; 9 Institute for Human Infection and Immunity, University of Texas Medical Branch, Galveston, TX 77555-0610, USA

**Keywords:** mosquito-borne viruses, dengue, vector–pathogen–host interactions, cutaneous immunity, human skin

## Abstract

The most prevalent arthropod-borne viruses, including the dengue viruses, are primarily transmitted by infected mosquitoes. However, the dynamics of dengue virus (DENV) infection and dissemination in human skin following *Aedes aegypti* probing remain poorly understood. We exposed human skin explants to adult female *Ae. aegypti* mosquitoes following their infection with DENV-2 by intrathoracic injection. Skin explants inoculated with a similar quantity of DENV-2 by a bifurcated needle were used as controls. Quantitative in situ imaging revealed that DENV replication was greatest in keratinocytes in the base of the epidermis, accounting for 50–60% of all infected cells regardless of the route of inoculation. However, DENV inoculation by *Ae. aegypti* probing resulted in an earlier and increased viral replication in the dermis, infecting twice as many cells at 24 h when compared to needle inoculation. Within the dermis, enhanced replication of DENV by *Ae. aegypti* infected mosquitoes was mediated by increased local recruitment of skin-resident macrophages, dermal dendritic cells, and epidermal Langerhans cells relative to needle inoculation. An enhanced but less pronounced influx of resident myeloid cells to the site of mosquito probing was also observed in the absence of infection. *Ae. aegypti* probing also increased recruitment and infection of dermal mast cells. Our findings reveal for the first time that keratinocytes are the primary targets of DENV infection following *Ae. aegypti* inoculation, even though most of the virus is inoculated into the dermis during probing. The data also show that mosquito probing promotes the local recruitment and infection of skin-resident myeloid cells in the absence of an intact vasculature, indicating that influx of blood-derived neutrophils is not an essential requirement for DENV spread within and out of skin.

## 1. Introduction

Arboviral (arthropod-borne viral) diseases represent a major threat to human and animal health worldwide [1,2]. Among the common hematophagous arthropod vectors, *Aedes spp.* mosquitoes are responsible for transmitting some of the most devastating and medically important viruses, such as dengue, Zika, chikungunya, and yellow fever viruses [2,3]. Globally, these viruses infect hundreds of millions of people each year, causing large-scale epidemics with significant morbidity and mortality [1,2,3,4,5]. The dengue viruses (DENV1-4) are the most widespread and important mosquito-transmitted viruses in terms of global health [4,5,6]. In 2023, DENV infections were responsible for over 4.5 million symptomatic cases in the Americas alone, and thousands of individuals showed serious clinical symptoms [7]. Importantly, the burden of *Aedes*-transmitted viral infections is predicted to increase with climate change and the associated increase in vector density and expansion of the geographical range of *Aedes* mosquitoes [4,5,8,9,10].

The transmission from an infected female *Aedes* mosquito to a human host is a critical event in the life cycle of DENV. Previous studies have shown that DENV rapidly replicates and spreads in cutaneous tissues following needle inoculation, suggesting that cell populations within the skin constitute a cellular substrate for viral amplification during flavivirus infection [11,12,13]. Several skin-resident immune cell populations are infected upon needle inoculation, including the mononuclear phagocyte subsets of Langerhans cells, macrophages, and dermal dendritic cells (DCs) [12,13,14,15,16,17,18,19,20,21,22]. Other non-immune skin-resident cells, including keratinocytes in the epidermis and fibroblasts in the dermis, are also permissive to DENV infection and contribute to the initial immune response against the virus [12,13,23]. Following needle inoculation, keratinocytes in the epidermis are the earliest and most abundant targets of DENV infection within human skin explants [12]. Infected keratinocytes orchestrate the movement of virus-permissive skin-resident myeloid cells to the site of inoculation by producing inflammatory cytokines and chemokines in response to infection, which ultimately contributes to the spread of the virus. However, during natural vector-borne transmission of the virus, the mosquito inserts its proboscis directly into the dermis where it releases saliva and virus during probing to seek a blood vessel [14]. It is not clear if there is substantial or indeed any DENV infection in the epidermis during this event; therefore, the role of infected keratinocytes in inflammation and myeloid cell recruitment during natural virus transmission is unknown.

*Ae. aegypti* saliva has a myriad of unique proteins that have antiplatelet, anticlotting, and vasodilatory properties, as well as modulators of inflammation, immunity, and angiogenesis [24,25]. These salivary factors have been shown to affect arboviral establishment in the host in numerous in vitro and murine studies [25,26,27,28,29,30,31,32,33,34]. Previous reports have shown that DENV delivered to mice by infected *Ae. aegypti* mosquitoes resulted in viremias with higher peak titers and longer duration compared to those infected by needle injection [28,29]. In another report, inoculation of Semliki Forest virus in a skin site previously pierced by *Ae. aegypti* resulted in higher viremia and increased mortality in rodents [35]. Even in the absence of virus, mosquito piercing can induce skin edema and an influx of neutrophils from blood that promotes the recruitment of virus-permissive myeloid cells to the skin [35]. The probing of uninfected *Ae. aegypti* in human volunteers also leads to neutrophil influx and degranulation followed by the recruitment of skin-resident DCs and macrophages [36]. There is thus an apparent discrepancy between data generated using human skin explants, which reveal that the recruitment of skin-resident myeloid cells is a function of keratinocyte activation and the release of proinflammatory cytokines and chemokines, and in vivo data, which show that the critical event in virus infection and spread following mosquito transmission is the recruitment of neutrophils from blood.

In this study, we adapted an established ex vivo model of flavivirus infection of human skin [11,12] to define the early cutaneous events following the probing of DENV-infected and uninfected *Ae. aegypti* mosquitoes. We specifically addressed whether keratinocytes were primary and major targets of infection following mosquito probing, and whether probing provided any additional positive effects on myeloid cell recruitment over those induced by needle inoculation alone.

## 2. Materials and Methods

### 2.1. Study Approval

Surgically derived skin specimens from four healthy women from the Pittsburgh, Pennsylvania, region were used in this study. These individuals were undergoing elective panniculectomy or abdominoplasty after successful weight loss programs through the Department of Plastic Surgery at the University of Pittsburgh. All individuals gave informed consent for resected skin to be used for research purposes. Skin specimens were not collected specifically for this research, and investigators were not able to link specimens to any identifiable private information of donors. Thus, the study did not constitute human subject research and was exempt from review by the Institutional Review Board of the University of Pittsburgh.

### 2.2. Cells and Viruses

C6/36 cells (*Aedes albopictus* mosquito cell line; ATCC CRL-1660) were incubated at 28 °C in 5% CO_2_, while Vero cells (African green monkey kidney epithelial; ATCC CCL-81) were incubated at 37 °C in 5% CO_2_. Both cell lines were maintained in Dulbecco’s modified Eagle medium (DMEM) that was supplemented with 10% (*v*/*v*) heat-inactivated fetal bovine serum, 2 mM l-glutamine, 1.5 g/L sodium bicarbonate, 100 U/mL penicillin, and 100 μg/mL of streptomycin. C6/36 cell media was supplemented with 1% (*v*/*v*) tryptose phosphate broth. Virus stocks of the prototype DENV-2 (strain Thailand/16681/1964) serotype were acquired from the World Reference Center for Emerging Viruses and Arboviruses (WRCEVA). DENV-2 was propagated in C6/36 cells, titrated using a plaque assay with Vero cells [11], and then stored at –80 °C.

### 2.3. Skin Processing and Shipping

Abdominal skin specimens were recovered immediately post-surgery from four healthy women in the Pittsburgh, Pennsylvania, region undergoing elective panniculectomy. Lack of immunity to DENV-1–4 was confirmed by ELISA using sera collected from the subcutaneous blood capillary network. For skin processing, residual adipose tissue was trimmed from the underside of the skin immediately after surgery, and tissue was cut into full-thickness two square-inch explants. Explants were folded with the dermis side outermost and the resulting skin sandwich was wrapped in gauze soaked in nutrient media (RPMI 1640 medium supplemented with 10% fetal bovine serum, 2 mM l-glutamine, 100 U/mL penicillin, 100 μg/mL of streptomycin, 10 mM HEPES, 1% sodium pyruvate, and 1% nonessential amino acids) [37]. The wrapped skin was then placed into a primary container, which was surrounded by layers of non-frozen and frozen ice packs before being placed in a secondary container for overnight transportation at refrigeration temperature from the University of Pittsburgh to the University of Texas Medical Branch. Immediately after being received, explants were placed dermis-side down in tissue culture dishes and incubated at the liquid–air interface in nutrient media at 37 °C for 1 h for acclimation before experiments.

### 2.4. Mosquito Inoculation

We used legacy colonies of the laboratory-adapted Rockefeller *Aedes aegypti* strain [38]. The *Aedes aegypti* colony was reared using standard methods by the University of Texas Medical Branch Insectary Services Core. Four-day post-eclosion female *Ae. aegypti* mosquitoes were cold-anesthetized and inoculated with 8.6 × 10^2^ FFU DENV-2 in a volume of 0.2 μL, or an equal volume of Dulbecco’s Phosphate-Buffered Saline (PBS) by intrathoracic injection using a Nanoject II (Drummond Scientific, Broomall, PA, USA). Following inoculation, mosquitoes were maintained with regular access to water and 10% sucrose solution for 12 days before experiments.

### 2.5. DENV-2 Inoculation of Human Skin Explants

DENV-2-infected and control female mosquitoes were starved of sucrose overnight and deprived of water for a minimum of 6 h, prior to exposure to warmed human skin explants. To focus probing to the central region, the skin was covered with sterile filter paper exposing only the central 1-inch diameter region of skin. We used five mosquitoes per square inch of skin based on previous mouse (5 mosquitoes/footpad) [35] and macaque studies (10–50 mosquitoes/arm) [39]. Mosquitoes were allowed to probe the skin for 30 min in the central area of the specimen, which was taped off from the surrounding unprobed skin. After probing, the skin explant was repeatedly immersed in media containing antibiotics and fungicides to remove contaminants potentially introduced by the mosquitoes. Needle inoculation was carried out in parallel as previously described [11,12]. We placed a 50 μL suspension containing 1.1 × 10^4^ FFU of virus in the center of the skin explant. We then repeatedly puncture the skin surface using a bifurcated skin allergy testing needle (Röchling Medical) to deliver the virus into the epidermis and dermis. The maximum depth of penetration for the bifurcated needle (inner length of the bifurcated needle prong) is around 2.5 mm in length, which is comparable to the fascicle of *Ae. aegypti* mosquitoes (~1.5 to 2.5 mm in length) [40,41]. For both experimental conditions, we started with a large skin area (2-inch square explants) to allow for cellular recruitment to the area of viral inoculation. Mosquito- and needle-inoculated explants were incubated at 37 °C in 5% CO_2_ for 2 h. We then placed the skin explants on mesh grids with the dermis side down in tissue culture dishes. Explants were then incubated in nutrient media at 37 °C at the liquid–air interface for 2, 8, and 24 h.

### 2.6. Confocal Microscopy

Immunofluorescence staining was performed as previously described [11,12]. We used the following primary antibodies to stain for virus- and cell-specific markers: polyclonal rabbit anti-pan DENV NS3 (kindly provided by Sujan Shresta, La Jolla Institute for Allergy and Immunology, San Diego, CA, USA), monoclonal anti-cytokeratin pan type I (AE-1; Invitrogen, Waltham, MA, USA, MA5-13144, Thermo Fisher Scientific, Wilmington, DE, USA), anti-CD163 (5C6-FAT; BM4041, Novus Biologicals, Centennial, CO, USA), anti-CD207/langerin (DCGM4; IM3449, Beckman Coulter, Inc., Brea, CA, USA), anti-CD1c (L161; ab190305, Abcam, Cambridge, UK), anti-CD117 (104D2; 604-140, Thermo Fisher Scientific), and anti-FcεR (9E1; NB100-63269, Novus Biologicals). Secondary antibodies were from Invitrogen, Thermo Fisher Scientific, and included goat anti-mouse IgG1, Alexa Fluor 546 (A-21123), goat anti-mouse IgG2b, Alexa Fluor 647 (A21242), and donkey anti-rabbit IgG, Alexa Fluor 488 (A-21206). Slides were counterstained with Hoescht dye to identify cell nuclei. Images were viewed on an Olympus Fluoview 1000 confocal microscope (Olympus, Tokyo, Japan).

### 2.7. Quantitative Image Analysis

Image analysis was performed by thresholding for positive staining and normalizing to total tissue area using Nikon NIS-Elements AR 4.50.00 software (version 5.30.03), as previously described [11,12]. When quantifying cell density using cell-specific antibodies, only regions of fluorescence that overlapped with the nuclear dye and hence were cell-associated were included in the analysis. Regions of interest were drawn to delineate the epidermis and dermis and these regions were combined to give an overall total area of imaged skin for analysis. Stains of interest were quantified by recording the binary area as pixels. For each skin specimen, data were collected from a minimum of 10 confocal images taken from sections collected from different sites of the virus-inoculated tissue. Values from each section were averaged and presented as individual data points. Data are presented for 4 skin donors per experiment.

### 2.8. Statistical Analyses

Results are representative of four independent experiments and are expressed as mean ± SEM. Differences between 2 groups were assessed using an unpaired 2-tailed Mann–Whitney U test. Multiple comparisons were performed using a Kruskal–Wallis 1-way ANOVA followed by Dunn’s multiple comparisons test. The statistical analysis was conducted using GraphPad Prism software version 10.0a, and the significant level was set at 5%.

## 3. Results

### 3.1. DENV-2 Primarily Infects Keratinocytes Following the Probing of Infected Ae. aegypti Mosquitoes

**Figure 1:**
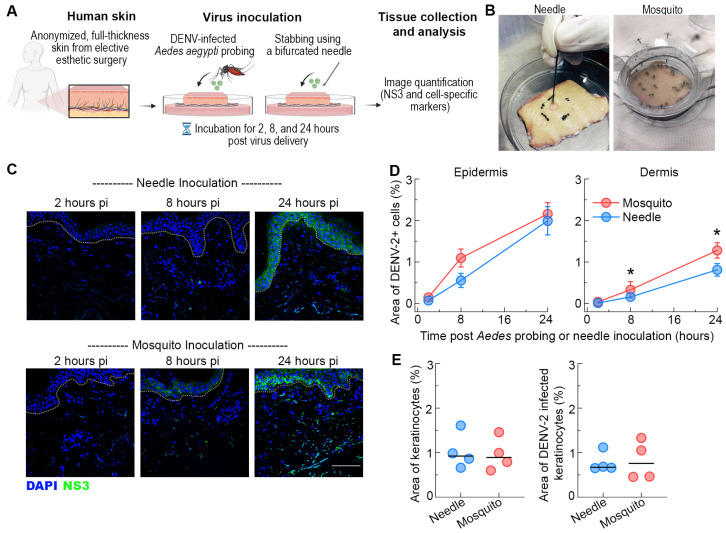
Similar infection in the epidermis but enhanced infection in dermis following *Ae. aegypti* inoculation of DENV-2. (**A**) Schematic representation of the experimental approach. (**B**) Images of skin explants inoculated via probing by puncturing using a bifurcated needle or by DENV-2-infected *Ae. aegypti* mosquitoes. (**C**) Representative images showing NS3 expression (green) at 2, 8, and 24 h after needle or mosquito inoculation in human skin. Blue staining in images represents nuclei and the dotted lines indicate the epidermal-dermal junction. Scale bar 50 μm. (**D**) Quantification of DENV-2 infection in the epidermis and dermis. Data are expressed as the area of NS3-positive cells as a percentage of the total imaged skin. Data are from 4 skin donors and expressed as mean ± SEM. * *p* < 0.05 (Mann–Whitney U test) comparing mosquito versus needle at each time point. (**E**) Quantification of the area of epidermal keratinocytes (AE1+) and area of infected keratinocytes (AE1+NS3+) in the skin after DENV-2 inoculation by needle or mosquito in human skin. Each symbol is an individual donor, and the horizontal line is the median.

We took advantage of our well-established ex vivo human skin model of flavivirus infection [11,12] to analyze the direct effects of mosquito probing on DENV infection and replication in human skin. We have previously shown that intrathoracic inoculation of female *Ae. aegypti* mosquitoes leads to between 10^2^ and 10^3^ focus-forming units (FFUs) of virus titer in mosquito saliva [42]. For comparison, we inoculated skin from the same donors by repeated puncture of the skin surface using a bifurcated needle with 1.1 × 10^4^ FFU, equivalent to the maximum virus dose inoculated by five mosquitoes, or PBS in a 1 cm^2^ area demarcated in the center of the skin (Figure 1A,B). Explants were then incubated at the liquid–air interface and collected at various intervals, as described [11,12].

We first examined the effect of *Ae. aegypti* probing on DENV infection and replication in the epidermis by staining for virus NS3 protein, which is expressed only during replication. Tissues were observed by confocal microscopy and the area of NS3 staining was quantified by image analysis after normalizing for the total area of tissue examined. NS3 expression was detected in the epidermis as early as 8 h post-infection, and replicating virus disseminated through much of the epidermis within 24 h. The extent of DENV infection in this skin compartment was similar between *Ae. aegypti*-probed or needle-inoculated skin (Figure 1C,D). Previous studies using needle inoculation demonstrate that keratinocytes account for the majority of the DENV-infected cells in the epidermis [12]. To confirm that the profile of *Ae. aegypti* probe-infected cells in the epidermis was similar to that of the needle inoculation, we stained skin sections with AE1 (to label for keratinocytes) in addition to NS3. Quantitative image analysis showed DENV infection of keratinocytes in both the *Ae. aegypti*-probed and needle-inoculated skin and revealed no differences in the density of infected keratinocytes (AE1+NS3+ cells) between inoculation conditions at 24 h (Figure 1E). These findings further reinforce the importance of keratinocytes in natural DENV infection of humans [11,12,23,34,43].

We next focused on the effect of mosquito probing on DENV infection in the dermal layer. In contrast to the epidermis, infection with DENV-2 by *Ae. aegypti* probing resulted in a substantial increase in the area of NS3 staining in the dermis when compared to needle inoculation (Figure 1C). This effect was detected as early as 8 h and was most pronounced at 24 h post-infection (Figure 1D). At this timepoint, DENV-2 infection by *Ae. aegypti* probing resulted in a ~2-fold increase in the density of infected cells in the dermis than needle inoculation. These results suggest that DENV replicates and disseminates more efficiently within the dermal layer following mosquito probing.

### 3.2. Ae. aegypti Probing Increases the Recruitment of Myeloid Cells within the Dermis of Skin Inoculated with DENV-2

**Figure 2:**
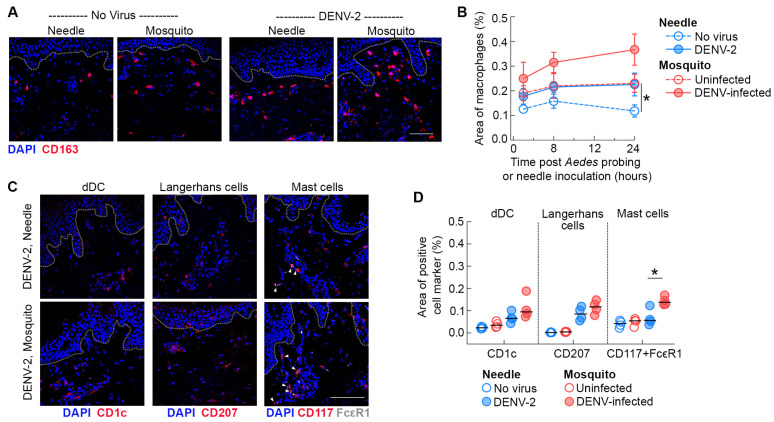
*Ae. aegypti* probing increases the recruitment of resident myeloid cells in the dermis of skin inoculated with DENV-2. (**A**) Immunofluorescence of skin stained with antibodies against CD163 (macrophages, red) 24 h after needle or mosquito inoculation of DENV-2 in human skin. Blue staining represents nuclei and dotted lines indicate epidermal–dermal junction. Scale bar 50 μm. (**B**) Quantification of the density of macrophages in mock-infected skin (open symbols) and infected skin (closed symbols) over time. Data are from 4 skin donors expressed as mean ± SEM. * *p* < 0.05 (Mann–Whitney U test) comparing uninfected mosquito versus mock-needle at 24 h. (**C**) Immunofluorescence of skin stained with antibodies against CD1c (dermal DCs, red), CD207 (Langerhans cells, red), and CD117 and FcεR (mast cells, red and white) 24 h after needle or mosquito inoculation of DENV-2 in human skin. Blue staining represents nuclei and dotted lines indicate epidermal–dermal junction. Arrowheads indicate double staining for CD117 and FcεR in mast cells. Scale bar 50 μm. (**D**) Quantification of the density of dermal dendritic cells, Langerhans cells, and mast cells at 24 h under different conditions. Each symbol is an individual donor, and the horizontal line is the median. * *p* < 0.05 (Mann–Whitney U test) comparing DENV-2 infected mosquito versus needle.

To determine whether myeloid cell recruitment accounted for the increased area of infection within the dermis, we first quantified the density of macrophages, identified by the expression of CD163, in skin inoculated in the absence or presence of mosquito probing (Figure 2A). In DENV-2 infected skin, increased recruitment of macrophages by either *Ae. aegypti* probing or needle was observed as early as 2 h post-virus inoculation. Notably, DENV-2 infection of the skin by *Ae. aegypti* probing more than doubled the density of macrophages within the dermis relative to DENV-2 infection by needle at 24 h post-inoculation. We also found that *Ae. aegypti* probing of skin by uninfected mosquitoes boosted macrophage recruitment to the probing site to the same extent as that seen in skin infected with DENV-2 by needle inoculation (Figure 2B).

To further explore whether this effect extends to other skin-resident myeloid cells, we stained skin sections with antibodies to label dermal DCs (CD1c) and Langerhans cells (CD207) (Figure 2C). We found a modest but not significant increase in the density of dermal DCs in the dermis of skin infected with DENV-2 by *Ae. aegypti* probing relative to needle-inoculated skin, likely due to the small sample size. Quantitative image analysis also revealed an increased trend in the density of Langerhans cells within the dermis of skin inoculated by DENV-infected *Ae. aegypti* mosquitoes, reflecting enhanced migration of this cell subset from the epidermal to the dermal layer. In addition, we found a significant increase in the density of mast cells (identified by co-expression of CD117 and FcεR) within the dermis of DENV-2 infected skin by *Ae. aegypti* probing relative to needle inoculation (Figure 2C) observed in our study. Collectively, our findings suggest that *Ae. aegypti* probing has a substantial impact on the recruitment of skin-resident myeloid cells to the inoculation site following DENV-2 infection in human skin.

### 3.3. Ae. aegypti Probing Enhances DENV-2 Infection of Myeloid Cells in the Dermis of Human Skin

**Figure 3:**
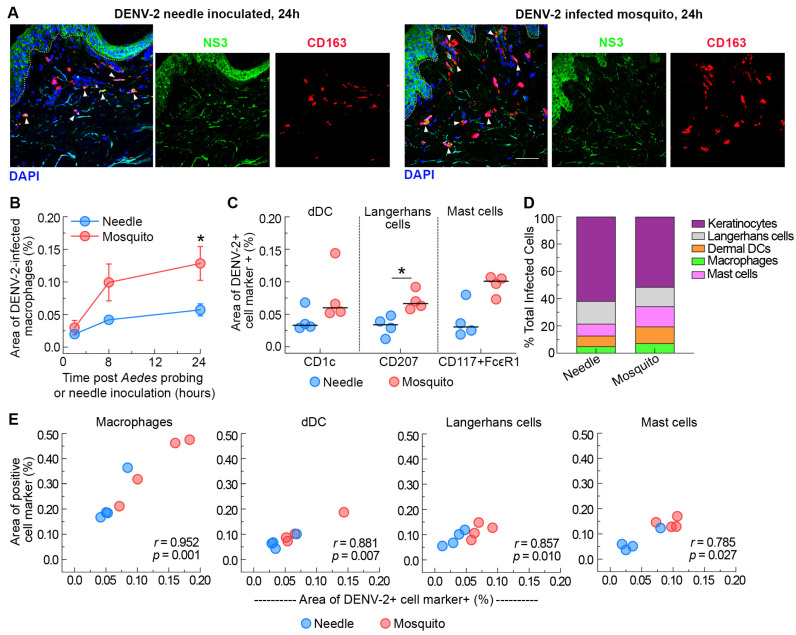
*Ae. aegypti* probing enhances DENV-2 infection of resident myeloid cells in the dermis of human skin. (**A**) Immunofluorescence of skin stained with antibodies against CD163 (macrophages, red) and NS3 (green) 24 h after needle or mosquito inoculation of DENV-2 in human skin. Blue staining represents nuclei and dotted lines indicate epidermal–dermal junction. Arrowheads indicate dengue-infected macrophages. Scale bar 50 μm. (**B**) Quantification of the density of infected macrophages (CD163+NS3+) after needle or mosquito inoculation of DENV-2 in human skin over time. Data are from 4 skin donors expressed as mean ± SEM. * *p* = 0.05 (Mann–Whitney U test) comparing DENV-2-infected mosquito versus needle. (**C**) Quantification of the density of infected dermal dendritic cells (CD163+NS3+), Langerhans cells (CD207+NS3+), and mast cells (CD117+FcεR+NS3+) at 24 h after needle or mosquito inoculation of DENV-2 in human skin. Each symbol is an individual donor, and the horizontal line is the median. * *p* < 0.05 (Mann–Whitney U test) comparing DENV-2-infected mosquito versus needle. (**D**) Infection as a percentage of all infected cells at 24 h after needle or mosquito inoculation of DENV-2 in human skin. Data represent the average of 4 skin donors. (**E**) Relationship between cell density and DENV-2 infection for each cell type.

We next examined the contribution of mosquito probing to the infection of myeloid cells with DENV-2. We found that *Ae. aegypti* probing increased the density of infected macrophages 2- to 3-fold at 8 and 24 h post-infection, respectively, relative to needle-inoculated skin (Figure 3A,B). Quantitative image analysis revealed that ~35% of the macrophages were DENV-2-positive in the *Ae. aegypti*-inoculated skin compared to 25% in the needle-inoculated skin (*p* = 0.028) at 24 h post-infection. In addition, there was a 2-fold increase in the density of infected dermal DCs and Langerhans cells relative to needle-inoculated skin at 24 h post-infection (Figure 3C). Of note, the increased recruitment of infected Langerhans cells to the dermis following mosquito probing observed at 24 h post-inoculation likely explains the transient differences in the overall density of infected cells in the epidermal layer in mosquito- versus needle-inoculated skin (Figure 1D). A previous report showed productive infection of mast cells when DENV-infected *Ae. aegypti* were allowed to feed on biopsy punches of human skin tissues [22]. We confirm and expand this finding by showing that *Ae. aegypti* probing significantly enhances the density of DENV-2 infected mast cells within the dermis of mosquitoes relative to needle-inoculated skin (Figure 3C). We also found a strong positive relationship between the recruitment of macrophages, dermal dendritic cells, Langerhans cells, and mast cells and their infection with DENV-2 (Figure 3E). In addition, myeloid cells made up roughly 50% of all DENV-infected cells at 24 h post-infection by *Ae. aegypti* probing, while in needle-inoculated skin around 40% of the infected cells were myeloid cells at the same time point (Figure 3D). Collectively, our data suggests that *Ae. aegypti* mosquito probing increases local recruitment of virus-susceptible myeloid cells to the foci of infection, even without an intact blood supply to the skin.

## 4. Discussion

Our findings reveal that DENV delivered via infected *Ae. aegypti* mosquitoes infect keratinocytes in the basal epidermis of human skin, despite probing occurring within the dermis. This is consistent with earlier reports indicating that epidermal cells are amongst the initial targets of DENV when experimentally inoculated via a needle in human skin [11,12,13]. Whether viral infection of epidermal cells occurs when the mosquito proboscis penetrates the epidermis or during active probing in the dermis is not known. However, the predominance of infection in the base of the epidermis at the epidermal–dermal junction is consistent with the virus infecting basal keratinocytes following deposition in the dermis.

Keratinocytes make up around 90% of the epidermal cells and contribute significantly to DENV infection in this layer [12]. Interestingly, we found no differences in the density of infected keratinocytes (AE1+NS3+ cells) between mosquito probing and needle-inoculated skin at 24 h. This is in contrast with earlier in vitro reports showing enhanced DENV replication in primary human epidermal keratinocytes infected in the presence of *Ae. aegypti* salivary gland extracts or isolated saliva proteins [34,43]. The epidermal–dermal immunological crosstalk likely impacts the dynamics of viral infection and dissemination to different compartments in intact human skin, which likely accounts for the differences observed in our study with skin explants and studies using isolated cells. In addition, differences in the amount of saliva used between experimental systems might also account for these discrepancies, as only a few nanoliters of saliva are injected during mosquito probing.

Our data on vector–virus–host interaction in the ex vivo human skin model indicates that *Ae. aegypti* probing greatly enhances DENV infection and replication within the dermis. This finding is in line with previous in vitro and murine studies showing enhanced arboviral infection in the presence of mosquito saliva [28,29,30,32,33,34,35,43,44]. Mosquito feeding consists of two discrete phases, probing and engorgement [14]. It is during the probing phase that saliva and by association virus is injected into the skin. In our experimental system, engorgement does not occur, as the skin is devoid of an intact vasculature and without blood pressure the dermal vessels lack blood. When compared to needle inoculation, co-inoculation of DENV with *Ae. aegypti* saliva, feeding of uninfected *Ae. aegypti* mosquitoes before DENV injection, or inoculation by DENV-infected *Ae. aegypti* mosquitoes have been associated with higher and more sustained viremia, increased viral titers in the skin, worse clinical symptoms, and dengue pathogenesis in several murine models [27,28,29,32,33]. Our data build upon these earlier animal studies and confirm that mosquito-derived factors also enhance DENV replication at the site of viral transmission in humans. Of note, due to the similarities in midgut bacterial microbiome [45], we would expect similar results in skin inoculated using geographically and genetically diverse *Ae. aegypti* laboratory colonies.

In line with earlier reports [29,32,33,46], we found a significant boost in the density of macrophages, dermal DCs, and Langerhans cells in the dermis of skin infected with DENV-2 by *Ae. aegypti* probing relative to needle-inoculated skin, despite only having four pairs of human skin specimens for analysis. This is consistent with studies showing that mosquito probing triggers the activation of pro-inflammatory genes in response to biting and virus sensing in the skin, which contributes to the localized increase in immune cell migration post-probing [29,32,33,35,46]. In fact, *Ae. aegypti* probing of skin by uninfected mosquitoes also led to a boost in myeloid cell recruitment to the inoculation site. This finding is similar to the observation in a recent controlled human challenge showing that uninfected *Ae. aegypti* probing enhances the transcription of inflammatory genes and increases the proportion of macrophages, dermal DCs, and neutrophils to the skin post-probing [36].

In murine models, mosquito probing has been shown to enhance the recruitment of neutrophils and monocytes to the dermis and increase dendritic cell migration to skin-draining lymph nodes [29,32,33,35,46]. Studies with dengue and other arboviruses suggest that the increased recruitment of myeloid cells results from pro-inflammatory factors derived largely from the rapid influx of neutrophils observed shortly after mosquito probing [33,35,47,48]. Our data show that mosquito probing boosts the local recruitment of myeloid cells from the outer area of the tissue even in the absence of a neutrophil-driven inflammatory response. Although the source of pro-inflammatory factors following mosquito probing in the skin needs to be determined, chemokines produced by infected epidermal keratinocytes likely contribute to the increased cutaneous cellular trafficking, as shown in our previous studies [12].

The increased recruitment and infection of myeloid cells within the dermis of *Ae. aegypti*-probed skin observed in our study is consistent with previous reports showing that *Ae. aegypti* saliva suppresses the transcription of key viral detection genes and promotes inflammation in murine studies [29,32], which ultimately contribute to enhancing viral infectivity following mosquito probing. Mast cell degranulation following mosquito piercing has also been shown to regulate immune cell migration to the site of blood feeding [49], which is consistent with the boost in the density of mast cells within the dermis of DENV-2-infected skin by *Ae. aegypti* probing relative to needle inoculation found in our study. In addition, DENV infection of mast cells results in cell degranulation and the release of histamine and other mediators, which leads to vasodilation, fluid accumulation, and immune cell influx [22,50]. Even though we did not measure mast cell degranulation, the enhanced infection of mast cells following mosquito probing seen in our study likely boosts this effect and contributes to the increased recruitment of virus-susceptible myeloid cells to the inoculation site. This enhanced cellular trafficking likely contributes to augmenting the proportion of susceptible cells to the site of mosquito probing.

The ex vivo human skin system has some inherent limitations, the primary one being the lack of an intact vascular system. It is known that in intact skin, neutrophils and monocytes are recruited to the site of viral inoculation from blood, which clearly cannot occur with explants. While we observe increased local cell recruitment and virus infection following mosquito probing of skin explants as compared to needle inoculation, it is likely that neutrophil recruitment from blood in the skin with an intact vasculature would further enhance dengue infection in mosquito-probed skin, as seen in previous murine studies [25,28,29,33]. In addition, despite their relatively low concentration in the interstitial space, antibodies to mosquito salivary components or to previous dengue infections might also affect the observed results. Of note, our skin donors are from the Pittsburgh region, where exposure to *Ae. aegypti* mosquitoes and dengue virus are unlikely to occur [51]. Further studies, ideally using human skin explants from individuals living in endemic regions and infected by wild-caught mosquitoes, are needed to determine whether repeated exposure to *Ae. aegypti* probing and multiple dengue infections have a similar effect on DENV replication in human skin. The process of elective panniculectomy involves surgical resection of skin, which is expected to induce an inflammatory response at the surgical margins which would not be present at the time of a mosquito bite of intact skin. To limit this effect, we collected skin specimens within 4 h of resection and made inoculations at least 2 inches away from the surgical margin. We were also careful to maintain specimens at 4 °C during shipping. Previous studies have shown that expression of inflammatory cytokines and chemokines is markedly increased in skin following DENV inoculation; hence, the inherent effect of surgical resection does not mask the effects of virus infection on the inflammatory response [12]. Lastly, there are differences in mosquito landing behavior and probing in humans depending on the skin anatomy. Our skin samples were recovered from the abdomen, but *Ae. aegypti* feeding activity is slightly concentrated on the legs and forearms of standing volunteers (~50% below knees), but uniformly distributed among sleeping volunteers (23.3% below knees) [52]. While differences in the thickness of the epidermis and sensory sensitivities are seen in the palms of the hands and feet, the body parts preferred by mosquitoes in both standing or sleeping positions (forearms, legs, and abdomen) do not differ in terms of structure and immune cell composition of the skin [53].

## 5. Conclusions

In summary, our findings reveal that DENV delivered via probing of infected *Ae. aegypti* mosquitoes infect keratinocytes in the basal epidermis and enhance DENV infection and replication of resident myeloid cells in human skin, the primary site of arboviral transmission. This is notable because skin explants lack blood circulation; hence, myeloid cell recruitment and infection occur without an influx of neutrophils. Future studies are needed to clearly elucidate the mechanistic basis by which *Ae. aegypti* probing enhances DENV infection and modulates the cutaneous immune response, and how these early responses impact virus dissemination and pathogenesis. This knowledge is critical for the identification of mosquito saliva-derived targets that can lead to the clinical development of vector-targeted therapeutic strategies.

## Data Availability

Data are available upon request.
